# Survey data on perceived COVID-19 risk, COVID-19 vaccine perception, and COVID-19 vaccination intention among Vietnamese

**DOI:** 10.1016/j.dib.2022.107811

**Published:** 2022-01-11

**Authors:** Phi-Hung Nguyen, Duy Van Nguyen

**Affiliations:** a Faculty of Business, FPT University, Hanoi, Vietnam; b Department of Business Management, National Taipei University of Technology, Taipei, Taiwan; c QAglobal.co, Hanoi, Vietnam

**Keywords:** COVID-19, COVID-19 vaccine, Perceived COVID-19 risk, Vaccination intention, Vietnam

## Abstract

In the context of the COVID-19 response, this study presents an illustrated dataset to examine trust, COVID-19 risk perception, COVID-19 vaccination perception, subject norms, social media and intention to vaccinate among Vietnamese. Our questionnaire was conducted in Vietnamese and then translated into English and distributed to respondents through email and Facebook from June to July 2021, gathering 329 responses. Participation was voluntary, and participants were allowed to withdraw from the survey at any time. Data analysis was carried out using the SPSS 24.0 and Smart PLS 3.0 software packages following data cleansing and coding. The data summarizes respondents' socio-economic and demographic characteristics, and Statistical techniques were deployed to assess the validity and reliability of scales relating to COVID-19 vaccination intention in Vietnam. Additionally, these data will contribute to the existing literature about COVID-19 vaccination perceptions and intention to vaccinate among Vietnamese.

## Specifications Table

**Table:** 

Subject	Social Sciences (General)
Specific subject area	Epidemiology, Infectious diseases, Econometric analysis
Type of data	Table
How data were acquired	Survey questionnaire (Questionnaire included in Mendeley repository)
Data format	Raw, analyzed
Parameters for data collection	Respondents are randomly chosen for the survey, exclusively for subjects and vaccination intention against COVID-19.
Description of data collection	The survey was broadcast from 06/2021 to 07/2021 with the support of Internet platforms (Facebook, email, Google Form) and resulted in 329 responses.
Data source location	Region: Asia Country: Vietnam Location: Hanoi and Hochiminh city
Data accessibility	Repository name: Mendeley repository Data identification number: 10.17632/mz8krv3m5h.2 Direct URL to data: https://dx.doi.org/10.17632/mz8krv3m5h.2

## Value of the Data

• The data can ascertain what factors affect Vietnamese people before taking the COVID-19 vaccine.
• The data could assist researchers and professionals in determining the variables affecting people's intention to vaccinate against COVID-19, including Trust, risk perception, vaccine perception, and social influence.
• The data is helpful to identify individual characteristics influencing the COVID-19 vaccination intention based on respondents' socio-economic and demographic information.
• The data is topical when the vaccination program is implemented in Vietnam. Meanwhile, Vietnamese people still hesitate and lack information about the issue of the COVID-19 vaccine.

## 1 Data Description

Since the outbreak in Wuhan, China, in late 2019, the COVID-19 pandemic has spread worldwide, and it dangerously affects the health and economy world [1], [2]. Being a country sharing a border with China also affects Vietnam by the COVID-19 pandemic. Vietnam had to perform a nationwide lockdown in April 2020 and was socially isolated in some provinces and cities, with sudden increases in cases in 2020 and 2021. The peak of the COVID-19 pandemic in Vietnam to date is from 7/2021, with an outbreak of more than 4000 cases a day in Ho Chi Minh City. As of April 8, 2021, Vietnam has 174,461 infections and 2071 deaths. In June 2021, when the epidemic began to show signs of outbreak again, Vietnam started to administer vaccination in large numbers to localities with a sharp increase in the number of infections (although before that, Vietnam men have started to vaccinate local government agencies). Therefore, the study conducted questionnaire design and data collection to understand the citizen's awareness of the COVID-19 and COVID-19 vaccine, personal characteristics, and intention to vaccinate COVID-19 in Vietnam.

The survey is spread from 6/2021 to 7/2021 with the support of Internet platforms (Facebook, email, Google Form) and yielded 329 valid responses. The questionnaire is split into two sections: The first subdivision includes questions about trust, COVID-19 risk perception, COVID-19 vaccination perception, subject norm, social media and intention to acquire a COVID-19 vaccine. The second part gives information on respondents' demographic background.

The participant characteristics are presented in Table 1. The results show that male respondents accounted for 52.6% (173 people), women accounted for 45.6% (150 people), and other genders were 1.8% (6 people). The income is mainly ranged from 10 to 15 million VND/month (164 people, 49.8%). Of note, the study participants had a higher representation of participants under 35 years old (234 people, 73.9%). Half of the participants were private office staff (153 people, 46.5%). The majority had bachelor's degrees (242 people, 73.5%). The aspect of vaccination safety is one that people are concerned about while injecting the COVID-19 vaccine (147 people, 44.7%).

**Table 1:** Respondents' characteristics.

Respondents’ profiles (*N* = 329)	*N*	%		*N*	%
***Income (VND)***			***Gender***		
Under 10 million	51	15.5	Male	173	52.6
From 10 million to 15 million	164	49.8	Female	150	45.6
From 15 million to 20 million	45	13.7	Other	6	1.8
From 20 million or more	69	21	***Job***		
***Age***			Public Officials	27	8.2
35-45	1	0.3	Industrial workers	7	2.1
35-45 age	68	20.7	Self-employed	53	16.1
46-65 age	17	5.2	Private office staff	153	46.5
Under 35	243	73.9	Other	89	27
***Education***			***Indicators***		
University graduate	242	73.5	Reliable healthcare system	24	7.3
High school and below	21	6.4	No concern	67	20.4
Master	44	13.4	Vaccination safety	147	44.7
Doctor	22	6.7	Vaccination effectiveness	91	27.7

## 2 Experimental Design, Materials and Methods

The study used open-ended questions to screen the available information related to the COVID-19 vaccine perception in Vietnam. The open-ended questions included: ``Which criteria would you decide to take the COVID-19 vaccine?''; after getting answers and getting information about vaccine standards that people are interested in Vietnam, including: “Reliable healthcare system”; “Vaccination safety”; “Vaccination effectiveness”; “No concern”. Simultaneously, we discovered concerns with trust in the local healthcare system, the government's pandemic prevention efforts, and vaccination practices, impacting the COVID-19 vaccine perspective and individual mentalities. Therefore, the trust factor is also formed from reference to some previous studies [3] and interview results of the study (see items of Trust in Table 2).

**Table 2:** Reality analysis results.

Factors	Factor loading	Cronbach's Alpha
**Trust;***N* = **3, CR** = **0.926, AVE** = **0.677**		
Trust in the government's ability to prevent COVID-19.	0.806	0.904
Trust the vaccine being used by the Vietnamese government.	0.871	
Trust in the COVID-19 vaccine storage procedures.	0.843	
Trust in the medical team during the COVID-19 vaccination process.	0.840	
Trust in the ability to manage side effects after a COVID-19 vaccine.	0.813	
Trust that vaccines are the most effective disease prevention and control COVID-19.	0.761	

**Perceived COVID-19 Risk; *N*** = **4, CR** = **0.904, AVE** = **0.611**		
The COVID-19 pandemic has a high mortality rate.	0.819	0.873
Worrying about yourself, relatives, and colleagues who may be infected with COVID-19.	0.841	
Recognizing the possibility of a COVID-19 pandemic breaking out in the area where you live and work.	0.746	
Risk Perception of infection during concentrated isolation.	0.787	
Risk Perception of infection during self-isolation	0.710	
Risk perception of distance guidance during self-isolation.	0.781	

**COVID-19 Vaccine Perception; *N*** = **6, CR** = **0.957, AVE** = **0.788**		
Perceive that getting vaccinated against COVID-19 reduces the risk of the disease.	0.866	0.946
Perceive that getting vaccinated against COVID-19 reduces the severity of the disease.	0.850	
Perceive that vaccination against COVID-19 is required to prevent disease outbreaks.	0.885	
Perceive that vaccination against COVID-19 is good for the community.	0.916	
Perceive that vaccination against COVID-19 helps economic and social activities return to normal soon.	0.939	
Research on a COVID-19 vaccine is needed in the context of many new variants.	0.866	

**Subject Norm; *N*** = **3, CR** = **0.929, AVE** = **0.813**		
Impact of family members on your decision to get the COVID-19 vaccine.	0.931	0.886
Impact of friends and colleagues on your decision to get the COVID-19 vaccine.	0.924	
In general, you are easily influenced by people around you about getting the COVID-19 vaccine.	0.848	

**Social Media; *N*** = **3, CR** = **0.907, AVE** = **0.765**		
Regularly find out information about the COVID-19 vaccine on social networks.	0.890	0.847
Refer to the information shared from people who have received the COVID-19 vaccine on social networks.	0.883	
Social networks bring much helpful information to you about the COVID-19 vaccine.	0.850	

**Vaccination Intention; *N*** = **3, CR** = **0.888, AVE** = **0.729**		
Registered for the COVID-19 vaccine.	0.695	0.812
Expect to get a COVID-19 vaccine at any time.	0.929	
Ready to encourage loved ones to get vaccinated against COVID-19.	0.917	

Next section, this study is based on numerous prior studies to offer a more extensive perception of the COVID-19 risk and COVID-19 vaccination perception. The perceived COVID-19 risk is provided through six items derived from earlier research [4], [5], each of which reflects concern about the COVID-19 outbreak and its propagation to oneself and others. The COVID-19 vaccination perception is composed of six items adapted from earlier research [6] and responses from participants that indicated cognitive elements of the COVID-19 vaccine. The COVID-19 vaccination perception highlights people's positive attitudes toward the vaccine's efficacy (see items of the COVID-19 vaccine perception in Table 2).

The variables of the theory planned behavior (TPB) model are referenced and determined by polling respondents regarding the question's content. Subject norms and social media were shown to be associated with getting the COVID-19 vaccination. The items on these two factors were adapted from the research mentioned above [7], [8], [9] and re-adjusted to fit the COVID-19 vaccination scenario (see subject norm and social media items in Table 2).

It is nearly hard to deliver a survey directly in the circumstances of a severe outbreak of the COVID-19 pandemic in Vietnam. As a result, the survey was created and distributed online via social media platforms such as Facebook and email. The poll will run from June through July 2021. This is when Vietnam begins to re-epidemic and initiates vaccination in epidemic regions. At the closing of the survey, we collected 329 respondents for data analysis.

The convergence and reliability of the variables are investigated in this study. Cronbach's alpha has a widely accepted lower bound of 0.5. Furthermore, the factor loading value is determined from latent variables and the reliability coefficient via each item.

The results indicate that the factor loading coefficient ranges between 0.695 and 0.939, which is greater than 0.5, and the Average Variance Extracted (AVE) value is between 0.661 and 0.813, which is greater than 0.5, suggesting that all factors have converged. Additionally, Cronbach's Alpha coefficients range from 0.812 to 0.946, and Composite Reliability (CR) values for all scales were higher than 0.7 ranges from 0.904 to 0.957, meaning that all factors are reliable (see Table 2).

Discriminant validity was determined by comparing the square root of the AVE and correlation coefficients. The square root of AVE ranges between 0.782 and 0.902, all of which are greater than the equivalent correlation coefficient. This finding demonstrates that the variables are discriminating (see Table 3).

**Table 3:** Discriminant validity test.

	INT_	PCV	PV	SN	SO	TR
INT	0.854					
PCV	0.574	0.782				
PV	0.750	0.690	0.887			
SN	0.418	0.498	0.436	0.902		
SO	0.566	0.620	0.644	0.578	0.875	
TR	0.635	0.607	0.663	0.396	0.546	0.823

## Ethics Statement

This survey data was ethically reviewed by the FPT University's ethics committee (No. 1342/QD-DHFPT, 2021). The survey data was collected in accordance with the Helsinki Declaration. Respondents' participation was entirely voluntary, anonymous, and consensual.

## CRediT authorship contribution statement

**Phi-Hung Nguyen:** Conceptualization, Methodology, Supervision, Writing – review & editing, Resources. **Duy Van Nguyen:** Data curation, Formal analysis, Writing – original draft.

## Declaration of Competing Interest

The authors state that they have no financial interest or a competitive personal relationship in this article.

## Appendix Supplementary Materials

Image, application 1

Image, application 2

Image, application 3

Image, application 4

Image, application 5
