# Supplementary material for: Survey data on perceived COVID-19 risk, COVID-19 vaccine perception, and COVID-19 vaccination intention among Vietnamese
Source: Data Brief. 2022 Jan 11;40:107811. doi: 10.1016/j.dib.2022.107811 (PMC8747774; doi:10.1016/j.dib.2022.107811)
Supplement: Supplementary file 2 [file mmc2.docx]

**INFORMED CONSENT AND QUESTIONNAIRE**

1. **INFORMED CONSENT (BLANK FORM)**

Greetings, Sir/Madame

The advent of SARS-CoV-2 diseases necessitates an examination of immunization rates. However, few previous studies have looked into people's attitudes toward the COVID-19 vaccine in Vietnam and the factors influencing vaccination intentions.

This study aims to collect information about general individual concerns about the COVID-19 severity perception, perceived trust in government intervention strategy towards COVID-19 outbreak, COVID-19 vaccination perception, and intention to get vaccinated. This study was promoted by the Degree No 1097/QD-DHFPT from FPT University, Vietnam.

All your opinions are helpful in the research team, and none of them are considered right or wrong. Your personal information (if any) is kept confidential.

Do you agree to participate in the study? [ ] I agree [ ] I do not agree

| **STRUCTURE OF THE QUESTIONNAIRE** | | **PAGES** |
| --- | --- | --- |
| **Part 1** | **Questions about the content** | **2-3** |
| **Part 2** | **Personal information** | **4** |
| **Total pages** | | **4** |

Hanoi, 2021

**PART 1: QUESTIONS ABOUT THE CONTENT**

**A. Which criteria would you decide to take the COVID-19 vaccine?’**

| a. Trust in healthcare system | b. Vaccination safety |
| --- | --- |
| c. Vaccination effectiveness | d. No concern |

**B. Please tick your level of trust with the statements stated in the questions below according to the following levels:**

***1 = Completely distrustful; 2 = Do not trust; 3 = Ordinary; 4 = Trust; 5 = Completely Trust***

| **Code** | **Content** | **Level** | | | | |
| --- | --- | --- | --- | --- | --- | --- |
| 1. **Trust** | | | | | | |
| TR1 | Trust in the government's ability to prevent COVID-19. | 1 | 2 | 3 | 4 | 5 |
| TR2 | Trust the vaccine being used by the Vietnamese government. | 1 | 2 | 3 | 4 | 5 |
| TR3 | Trust in the COVID-19 vaccine storage procedures. | 1 | 2 | 3 | 4 | 5 |
| TR4 | Trust in the medical team during the COVID-19 vaccination process. | 1 | 2 | 3 | 4 | 5 |
| TR5 | Trust in the ability to manage side effects after a COVID-19 vaccine. | 1 | 2 | 3 | 4 | 5 |
| TR6 | Trust that vaccines are the most effective method of disease prevention and control COVID-19. | 1 | 2 | 3 | 4 | 5 |

**C. Please tick your level of agreement with the statements stated in the questions below according to the following levels:**

***1 = Totally disagree; 2 = Disagree; 3 = Neutral; 4 = Agree; 5 = Totally agree***

| **Code** | **Content** | **Level** | | | | |
| --- | --- | --- | --- | --- | --- | --- |
| 1. **Perceived COVID-19 Risk** | | | | | | |
| PRC1 | The COVID-19 pandemic has a high mortality rate. | 1 | 2 | 3 | 4 | 5 |
| PRC2 | Worrying about yourself, relatives, and colleagues who may be infected with COVID-19. | 1 | 2 | 3 | 4 | 5 |
| PRC3 | Recognizing the possibility of a COVID-19 will pandemic breaking out in the area where you live and work. | 1 | 2 | 3 | 4 | 5 |
| PRC4 | Risk Perception of infection during concentrated isolation. | 1 | 2 | 3 | 4 | 5 |
| PRC5 | Risk Perception of infection during self-isolation | 1 | 2 | 3 | 4 | 5 |
| PRC6 | Risk perception of distance guidance during self-isolation. | 1 | 2 | 3 | 4 | 5 |
| 1. **COVID-19 Vaccine Perception** | | | | | | |
| PV1 | Perceive that getting vaccinated against COVID-19 reduces the risk of the disease. | 1 | 2 | 3 | 4 | 5 |
| PV2 | Perceive that getting vaccinated against COVID-19 reduces the severity of the disease. | 1 | 2 | 3 | 4 | 5 |
| PV3 | Perceive that vaccination against COVID-19 is required to prevent disease outbreaks. | 1 | 2 | 3 | 4 | 5 |
| PV4 | Perceive that vaccination against COVID-19 is good for the community. | 1 | 2 | 3 | 4 | 5 |
| PV5 | Perceive that vaccination against COVID-19 helps economic and social activities return to normal soon. | 1 | 2 | 3 | 4 | 5 |
| PV6 | Research on a COVID-19 vaccine is needed in the context of many new variants. | 1 | 2 | 3 | 4 | 5 |
| 1. **Subjective Norm** | | | | | | |
| SN1 | Impact of family members on your decision to get the COVID-19 vaccine. | 1 | 2 | 3 | 4 | 5 |
| SN2 | Impact of friends and colleagues on your decision to get the COVID-19 vaccine. | 1 | 2 | 3 | 4 | 5 |
| SN3 | In general, you are easily influenced by people around you about getting the COVID-19 vaccine. | 1 | 2 | 3 | 4 | 5 |
| 1. **Social Media** | | | | | | |
| SM1 | Regularly find out information about the COVID-19 vaccine on social networks. | 1 | 2 | 3 | 4 | 5 |
| SM2 | Refer to the information shared from people who have received the COVID-19 vaccine on social networks. | 1 | 2 | 3 | 4 | 5 |
| SM3 | Social networks bring a lot of useful information to you about the COVID-19 vaccine. | 1 | 2 | 3 | 4 | 5 |
| 1. **Vaccination Intention** | | | | | | |
| INT1 | Registered for the COVID-19 vaccine. | 1 | 2 | 3 | 4 | 5 |
| INT2 | Expect to get a COVID-19 vaccine at any time. | 1 | 2 | 3 | 4 | 5 |
| INT3 | Ready to encourage loved ones to get vaccinated against COVID-19. | 1 | 2 | 3 | 4 | 5 |

**PART 2: PERSONAL INFORMATION**

Please provide information by ticking the corresponding box below:

| 1.Gender: | **❒** Male | **❒** Female |
| --- | --- | --- |
|  | | |
| 2. Age | **❒** Under 35 | **❒** 35-45 |
|  | **❒** 46-65 | **❒** >65 |
|  | | |
| 3. Job | **❒** Public Officials | **❒** Private office staff |
|  | **❒** Industrial workers | **❒** Other |
|  | **❒** Self-employed |  |
|  |  |  |
| 4. Education | **❒** University graduate | **❒** Master |
|  | **❒** High school and below | **❒** Doctor |
|  |  |  |
| 5. Monthly Income | **❒** Under 10 million VND | **❒** From 15 million to 20 million VND |
|  | **❒** From 10 million to 15 million VND | **❒** Higher than 20 million VND |
|  |  |  |

**_________ End of the questionnaire, thank you very much for your help_______**
