# Supplementary material for: Survey data on perceived COVID-19 risk, COVID-19 vaccine perception, and COVID-19 vaccination intention among Vietnamese
Source: Data Brief. 2022 Jan 11;40:107811. doi: 10.1016/j.dib.2022.107811 (PMC8747774; doi:10.1016/j.dib.2022.107811)
Supplement: Supplementary file 3 [file mmc3.docx]

**BẢNG KHẢO SÁT**

Thưa anh/chị

Chúng tôi là nhóm nghiên cứu trường Đại học FPT đang nghiên cứu đề tài liên quan tới việc tiêm Vaccine COVID-19 ở Việt Nam. Nghiên cứu này nhằm đánh giá một cách khách quan về vấn đề tiêm Vaccine COVID-19 của người dân. Để hoàn thành nghiên cứu này, chúng tôi rất mong nhận được sự hỗ trợ của anh/chị bằng cách trả lời các câu hỏi dưới đây. Mọi ý kiến của anh/chị đều có ích cho nhóm nghiên cứu và không có ý kiến nào được xem là đúng hay sai. Các thông tin cá nhân (nếu có) của anh/chị đều được bảo mật. Nếu có bất kỳ thắc mắc gì về nội dung nghiên cứu, vui lòng liên hệ với chúng tôi qua email: [Hungnp30@fe.edu.vn/](mailto:Hungnp30@fe.edu.vn/)

**I. NỘI DUNG CÂU HỎI**

*1. Anh/chị lựa chọn tiêu chí nào khi tiêm phòng vaccine COVID-19? (chọn nhiều đáp án)*

1. Vaccine an toàn 2. Vaccine hiệu quả

3. Cơ sở y tế đảm bảo 4. Theo chỉ đạo của nhà nước (tiêm được là tốt)

*2. Anh/chị cho rằng khả năng bạn bị nhiễm COVID-19 là bao nhiêu:……. (từ 1-100%)*

1. 0 % - 20% 2. 20% - 40%

3. 40% - 60% 4. 60%- 100%

*3. Anh/chị đánh giá về mức độ nghiêm trọng khi bị nhiễm COVID-19:*

1.Hoàn toàn không nghiêm trọng 2.không nghiêm trọng

3.bình thường 4. Nghiêm trọng 5. Cực nghiêm trọng

*4. Loại vac xin mà bạn mong muốn được tiêm nhất?*

*1. AstraZeneca 2.Comirnaty của Pfizer/BioNTech*

*3.Moderna 4.SPUTNIK V*

*5.Vero Cell của Sinopharm 6.Janssen 7.Nano Covax ( Nanogen)*

Anh/chị vui lòng trả lời các câu hỏi dưới đây bằng cách khoanh tròn vào mức độ đồng ý của mình với các phát biểu cho sẵn. Trong đó: **1 = Hoàn toàn không tin trưởng; 2 = Không tin trưởng; 3 = Bình thường; 4 = Tin tưởng; 5 = Hoàn toàn tin tưởng**

| Mã | Nội dung | | | Mức độ | | | | | | | |  |
| --- | --- | --- | --- | --- | --- | --- | --- | --- | --- | --- | --- | --- |
| 1. **Sự tin tưởng** | | | | | | | | | | | |  |
| TR1 | Anh/chị tin tưởng vào chính phủ trong vấn đề phòng chống COVID-19? | | | 1 | | 2 | | 3 | | 4 | 5 |  |
| TR2 | Anh/chị tin tưởng vào loại vaccine được chính phủ áp dụng tiêm ở Việt Nam | | | 1 | | 2 | | 3 | | 4 | 5 |  |
| TR3 | Anh/chị tin tưởng vào quá trình bảo quản tiêm vaccine COVID-19? | | | 1 | | 2 | | 3 | | 4 | 5 |  |
| TR4 | Anh/chị tin tưởng vào đội ngũ y bác sĩ trong quá trình tiêm vaccine COVID-19? | | | 1 | | 2 | | 3 | | 4 | 5 |  |
| TR5 | Anh/chị tin tưởng vào việc xử lý phản ứng phụ sau khi tiêm vaccine COVID-19? | | | 1 | | 2 | | 3 | | 4 | 5 |  |
| TR6 | Anh/chị tin tưởng vaccine là giải pháp hiệu quả nhất trong phòng và chống COVID-19? | | | 1 | | 2 | | 3 | | 4 | 5 |  |
| 1. **Nhận thức rủi ro do COVID-19** | | | | | | | | | | | |  |
| PRC1 | | Anh/chị nhận thức đại dịch COVID-19 có tỷ lệ tử vong cao. | | | 1 | | 2 | | 3 | 4 | 5 |  |
| PRC2 | | Anh/chị lo lắng cho bản thân, người thân và đồng nghiệp của anh/chị có thể bị nhiễm COVID-19 | | | 1 | | 2 | | 3 | 4 | 5 |  |
| PRC3 | | Anh/chị nhận thấy khả năng đại dịch COVID-19 sẽ bùng phát tại khu vực tôi sinh sống và làm việc. | | | 1 | | 2 | | 3 | 4 | 5 |  |
| PRC4 | | Anh/chị lo lắng về rủi ro nhiễm bệnh trong quá trình cách ly tập trung | | | 1 | | 2 | | 3 | 4 | 5 |  |
| PRC5 | | Anh/chị lo lắng về rủi ro nhiễm bệnh trong quá trình tự cách ly | | | 1 | | 2 | | 3 | 4 | 5 |  |
| PRC6 | | Anh/chị nhận thức rủi ro về hướng dẫn chữa bệnh từ xa trong quá trình tự cách ly | | | 1 | | 2 | | 3 | 4 | 5 |  |
| 1. **Nhận thức về tiêm Vaccine COVID-19** | | | | | | | | | | | |  |
| PV1 | | Anh/chị nhận thấy tiêm Vaccine COVID-19 giảm rủi ro nhiễm bệnh | | | 1 | | 2 | | 3 | 4 | 5 |  |
| PV2 | | Anh/chị nhận thấy tiêm Vaccine COVID-19 giảm mức độ nghiêm trọng khi nhiễm bệnh | | | 1 | | 2 | | 3 | 4 | 5 |  |
| PV3 | | Anh/chị nhận thấy tiêm Vaccine COVID-19 là bắt buộc để phòng chống dịch | | | 1 | | 2 | | 3 | 4 | 5 |  |
| PV4 | | Anh/chị nhận thấy tiêm Vaccine COVID-19 tốt cho cộng đồng | | | 1 | | 2 | | 3 | 4 | 5 |  |
| PV5 | | Anh/chị nhận thấy tiêm Vaccine COVID-19 giúp các hoạt động kinh tế, xã hội sớm trở lại bình thường | | | 1 | | 2 | | 3 | 4 | 5 |  |
| PV6 | | Anh/chị cho rằng việc nghiên cứu Vaccine COVID-19 là cần thiết trong bối cảnh có nhiều biến thể mới | | | 1 | | 2 | | 3 | 4 | 5 |  |
| 1. **MẠNG XÃ HỘI** | | | | | | | | | | | |  |
| SM1 | | Anh/chị thường xuyên tìm hiểu thông tin về Vaccine COVID-19 trên mạng xã hội | | | 1 | | 2 | | 3 | 4 | 5 |  |
| SM2 | | Anh/chị tham khảo những thông tin được chia sẻ từ người đã tiêm Vaccine COVID-19 trên mạng xã hội | | | 1 | | 2 | | 3 | 4 | 5 |  |
| SM3 | | Mạng xã hội mang lại nhiều thông tin hữu ích cho anh/chị về Vaccine COVID-19 | | | 1 | | 2 | | 3 | 4 | 5 |  |
| 1. **Chuẩn chủ quan** | | | | | | | | | | | |  |
| SN1 | | Tác động của những người thân trong gia đình đối với quyết định tiêm Vaccine COVID-19 của anh/chị | | | 1 | | 2 | | 3 | 4 | 5 |  |
| SN2 | | Tác động của những bạn bè, đồng nghiệp đối với quyết định tiêm Vaccine COVID-19 của anh/chị | | | 1 | | 2 | | 3 | 4 | 5 |  |
| SN3 | | Nhìn chung thì anh/chị dễ bị tác động bởi những người xung quoanh về việc tiêm Vaccine COVID-19 | | | 1 | | 2 | | 3 | 4 | 5 |  |
| 1. **Lưỡng lự** | | | | | | | | | | | |  |
| HE1 | | Anh/chị lo ngại Vaccine COVID-19 loại mới nhiều rủi ro hơn loại cũ đã được kiểm chứng | | | 1 | | 2 | | 3 | 4 | 5 |  |
| HE2 | | Anh/chị lo ngại về phản ứng phụ của Vaccine COVID-19 | | | 1 | | 2 | | 3 | 4 | 5 |  |
| HE3 | | Anh/chị lo ngại về bệnh lý nền khi tiêm Vaccine COVID-19 | | | 1 | | 2 | | 3 | 4 | 5 |  |
| HE4 | | Anh/chị lo lắng bị lây nhiễm COVID-19 khi đi tiêm phòng Vaccine COVID-19 | | | 1 | | 2 | | 3 | 4 | 5 |  |
| HE5 | | Anh/chị lưỡng lự về việc tiêm Vaccine COVID-19 | | | 1 | | 2 | | 3 | 4 | 5 |  |
| 1. **Ý định tiêm vaccine** | | | | | | | | | | | |  |
| INT1 | | Anh/chị đã đăng kí tiêm vaccine COVID-19 | | | 1 | | 2 | | 3 | 4 | 5 |  |
| INT2 | | Anh/chị có mong muốn tiêm vaccine COVID-19 bất cứ lúc nào | | | 1 | | 2 | | 3 | 4 | 5 |  |
| INT3 | | Anh/chị sẵn sàng động viên người thân tiêm vaccine COVID-19 | | | 1 | | 2 | | 3 | 4 | 5 |  |
| **II. THÔNG TIN CÁ NHÂN**  Bạn vui lòng cung cấp thông tin bằng cách tick vào ô tương ứng dưới đây: | | | | | | | | | | | | |
| 1. Giới tính: **❒** Nam | | | **❒** Nữ | | | | | | | | | |
| 1. Độ tuổi:   **❒** Dưới 35 **❒** 35-45  **❒** 46-65 **❒** >65   1. Nghề nghiệp | | | | | | | | | | | | |
| **❒** Nhân viên Văn phòng **❒** Công chức/viên chức  **❒** Kinh doanh (tiểu thương, chủ doanh nghiệp) **❒** Công nhân  **❒** Khác | | | | | | | | | | | | |
| 1. Công việc của bạn có hay phải tiếp xúc với người lạ không?   **❒** Có **❒** Không   1. Học vấn   **❒** Dưới Đai học **❒** Đai học  **❒** Thạc sĩ **❒** Tiến sĩ   1. Bạn đã kết hôn?   **❒** Có **❒** Không   1. Thu nhập của bạn? | | | | | | | | | | | | |
| **❒** Dưới 10 triệu **❒** Từ 10 triệu đến dưới 15 triệu  **❒** Từ 15 triệu đến dưới 20 triệu **❒** Từ 20 triệu trở lên | | | | | | | | | | | | |
| 1. Bạn có bệnh lý nền liên quan không?   **❒** Có **❒** Không   1. Bạn có người thân bị tư vong vì COVID-19?   **❒** Có **❒** Không   1. Nơi cư trú (Điền tên tỉnh/thành phố): …………………………………………………. | | | | | | | | | | | | |

Xin chân thành cảm ơn!
