# Supplementary material for: Survey data on perceived COVID-19 risk, COVID-19 vaccine perception, and COVID-19 vaccination intention among Vietnamese
Source: Data Brief. 2022 Jan 11;40:107811. doi: 10.1016/j.dib.2022.107811 (PMC8747774; doi:10.1016/j.dib.2022.107811)
Supplement: Supplementary file 4 [file mmc4.docx]

**QUESTIONAIRE**

Greetings: Sir/Madame

We are a study group from FPT University researching a topic about COVID-19 vaccine vaccination in Vietnam. The goal of this study is to assess the issue of COVID-19 immunization among people honestly. We need your advice to finish this study, so kindly answer the following questions. We appreciate your time to help complete this questionnaire. The information collected is only used to serve scientific research purposes. We will not, in any circumstances, share your information with other individuals or organizations without your consensus. Please feel free to contact us by email at Hungnp30@fe.edu.vn/ (+84) 985509890 if you have any questions about content research

| **STRUCTURE OF THE QUESTIONNAIRE** | | | **PAGES** | |
| --- | --- | --- | --- | --- |
| **Part 1** | **Questions about the content** | **2-3** | |  |
| **Part 2** | **Personal information** | **4** | |  |
| **Total pages** | | | **4** | |

- We appreciate your time to help complete this questionnaire.
- The information collected is only used to serve scientific research purposes. We will not, in any circumstances, share your information with other individuals or organizations without your consensus.

Hanoi, 2021

**PART 1: QUESTIONS ABOUT THE CONTENT**

**A. Which criteria would you decide to take the COVID-19 vaccine?’**

| a. Reliable healthcare system | b. Vaccination safety |
| --- | --- |
| c. Vaccination effectiveness | d. No concern |

**B. Please tick your level of trust with the statements stated in the questions below according to the following levels:**

***1 = Completely distrustful; 2 = Do not trust that boss; 3 = Ordinary; 4 = Trust; 5 = Completely trusting***

| **Code** | **Content** | **Level** | | | | |
| --- | --- | --- | --- | --- | --- | --- |
| 1. **Trust** | | | | | | |
| TR1 | Trust in the government's ability to prevent COVID-19. | 1 | 2 | 3 | 4 | 5 |
| TR2 | Trust the vaccine being used by the Vietnamese government. | 1 | 2 | 3 | 4 | 5 |
| TR3 | Trust in the COVID-19 vaccine storage procedures. | 1 | 2 | 3 | 4 | 5 |
| TR4 | Trust in the medical team during the COVID-19 vaccination process. | 1 | 2 | 3 | 4 | 5 |
| TR5 | Trust in the ability to manage side effects after a COVID-19 vaccine. | 1 | 2 | 3 | 4 | 5 |
| TR6 | Trust that vaccines are the most effective method of disease prevention and control COVID-19. | 1 | 2 | 3 | 4 | 5 |

**C. Please tick your level of agreement with the statements stated in the questions below according to the following levels:**

***1 = Totally disagree; 2 = Disagree; 3 = Neutral; 4 = Agree; 5 = Totally agree***

| **Code** | **Content** | **Level** | | | | |
| --- | --- | --- | --- | --- | --- | --- |
| 1. **Perceived COVID-19 Risk** | | | | | | |
| PRC1 | The COVID-19 pandemic has a high mortality rate. | 1 | 2 | 3 | 4 | 5 |
| PRC2 | Worrying about yourself, relatives, and colleagues who may be infected with COVID-19. | 1 | 2 | 3 | 4 | 5 |
| PRC3 | Recognizing the possibility of a COVID-19 will pandemic breaking out in the area where you live and work. | 1 | 2 | 3 | 4 | 5 |
| PRC4 | Risk Perception of infection during concentrated isolation. | 1 | 2 | 3 | 4 | 5 |
| PRC5 | Risk Perception of infection during self-isolation | 1 | 2 | 3 | 4 | 5 |
| PRC6 | Risk perception of distance guidance during self-isolation. | 1 | 2 | 3 | 4 | 5 |
| 1. **COVID-19 Vaccine Perception** | | | | | | |
| PV1 | Perceive that getting vaccinated against COVID-19 reduces the risk of the disease. | 1 | 2 | 3 | 4 | 5 |
| PV2 | Perceive that getting vaccinated against COVID-19 reduces the severity of the disease. | 1 | 2 | 3 | 4 | 5 |
| PV3 | Perceive that vaccination against COVID-19 is required to prevent disease outbreaks. | 1 | 2 | 3 | 4 | 5 |
| PV4 | Perceive that vaccination against COVID-19 is good for the community. | 1 | 2 | 3 | 4 | 5 |
| PV5 | Perceive that vaccination against COVID-19 helps economic and social activities return to normal soon. | 1 | 2 | 3 | 4 | 5 |
| PV6 | Research on a COVID-19 vaccine is needed in the context of many new variants. | 1 | 2 | 3 | 4 | 5 |
| 1. **Subjective Norm** | | | | | | |
| SN1 | Impact of family members on your decision to get the COVID-19 vaccine. | 1 | 2 | 3 | 4 | 5 |
| SN2 | Impact of friends and colleagues on your decision to get the COVID-19 vaccine. | 1 | 2 | 3 | 4 | 5 |
| SN3 | In general, you are easily influenced by people around you about getting the COVID-19 vaccine. | 1 | 2 | 3 | 4 | 5 |
| 1. **Social Media** | | | | | | |
| SM1 | Regularly find out information about the COVID-19 vaccine on social networks. | 1 | 2 | 3 | 4 | 5 |
| SM2 | Refer to the information shared from people who have received the COVID-19 vaccine on social networks. | 1 | 2 | 3 | 4 | 5 |
| SM3 | Social networks bring a lot of useful information to you about the COVID-19 vaccine. | 1 | 2 | 3 | 4 | 5 |
| 1. **Vaccination Intention** | | | | | | |
| INT1 | Registered for the COVID-19 vaccine. | 1 | 2 | 3 | 4 | 5 |
| INT2 | Expect to get a COVID-19 vaccine at any time. | 1 | 2 | 3 | 4 | 5 |
| INT3 | Ready to encourage loved ones to get vaccinated against COVID-19. | 1 | 2 | 3 | 4 | 5 |

**PART 2: PERSONAL INFORMATION**

Please provide information by ticking the corresponding box below:

| 1.Gender: | **❒** Nam | **❒** Nữ |
| --- | --- | --- |
|  | | |
| 2. Age | **❒** Under 35 | **❒** 35-45 |
|  | **❒** 46-65 | **❒** >65 |
|  | | |
| 3. Job | **❒** Public Officials | **❒** Private office staff |
|  | **❒** Industrial workers | **❒** Other |
|  | **❒** Self-employed |  |
|  |  |  |
| 4. Education | **❒** University graduate | **❒** Master |
|  | **❒** High school and below | **❒** Doctor |
|  |  |  |
| 5. Monthly Income | **❒** Under 10 million | **❒** From 15 million to 20 million |
|  | **❒** From 10 million to 15 million | **❒** From 20 million or more |
|  |  |  |

**_________ End of the questionnaire, thank you very much for your help_______**
