# Supplementary material for: Survey data on perceived COVID-19 risk, COVID-19 vaccine perception, and COVID-19 vaccination intention among Vietnamese
Source: Data Brief. 2022 Jan 11;40:107811. doi: 10.1016/j.dib.2022.107811 (PMC8747774; doi:10.1016/j.dib.2022.107811)
Supplement: Supplementary file 5 [file mmc5.docx]

https://docs.google.com/forms/d/e/1FAIpQLSe205dCiv5_qV-4rE_eJsnlQKOUKrly8TLfqowuw0epJfcTsw/viewform?fbzx=5701105053867929752&pli=1
